# Surgery for Complete Vertical Rectus Paralysis Combined with Horizontal Strabismus

**DOI:** 10.1155/2014/828919

**Published:** 2014-05-04

**Authors:** Leilei Zou, Rui Liu, Yan Liu, Jing Lin, Hong Liu

**Affiliations:** ^1^Department of Ophthalmology, Eye and ENT Hospital, Fudan University, 83 Fenyang Road, Shanghai 200031, China; ^2^Key Laboratory of Myopia, Ministry of Health PR China, 83 Fenyang Road, Shanghai 200031, China; ^3^Shanghai Key Laboratory of Visual Impairment and Restoration, Fudan University, 83 Fenyang Road, Shanghai 200031, China

## Abstract

*Aims.* To report outcomes of the simultaneous surgical correction of vertical rectus paralysis combined with moderate-to-large angle horizontal strabismus. *Methods.* If a preoperative forced duction test was positive, antagonist muscle weakening surgery was performed, and then augmented partial rectus muscle transposition (APRMT) + partial horizontal rectus recession-resection was performed 2 months later. If a preoperative forced duction test was negative, APRMT + partial horizontal rectus recession-resection was performed. Antagonistic muscle weakening surgery and/or conventional recession-resection of the horizontal and/or vertical muscles of the contralateral eye was performed 2 months later, as needed. *Results.* Ten patients with a mean age of 22.3 ± 13.0 years were included and mean follow-up was 7.1 months. The mean vertical deviation that APRMT corrected was 21.4 ± 3.7 PD (prism diopter). The absolute deviation in horizontal significantly decreased from a preoperative value of 48.5 ± 27.4 PD to a value of 3.0 ± 2.3 PD 6 months postoperatively. The movement score decreased from a value of −5 ± 0 preoperatively to a value of −2.7 ± 0.8 at 6 months postoperatively. *Conclusion.* For patients with complete vertical rectus paralysis combined with a moderate- to-large angle of horizontal strabismus, combined APRMT and partial horizontal rectus recession-resection is safe and effective for correcting vertical and horizontal strabismus.

## 1. Introduction

In cases of strabismus caused by rectus paralysis, the paralytic muscles are not able to contract, and on examination the eye cannot pass the midline in the direction of the major action of the affected muscle. Conventional rectus recession-resection is often not effective for strabismus due to rectus paralysis [1–3], though large recessions or periosteal fixation sutures can be effective in some cases [4–6]. Rectus paralysis can also be corrected by transposition or Jensen procedure of the rectus adjacent to the paralytic muscle [7–11]. There have been many attempts to improve the surgical management of strabismus due to rectus paralysis. Knapp [12] proposed the transposition of the 2 recti adjacent to the paralyzed muscle to the bilateral insertion of the paralyzed muscle to augment the eye movement in the direction of action of the paralytic muscle. Subsequent modifications divided the 2 recti adjacent to the paralyzed muscle into halves, and one-half was transposed to the insertion of the paralyzed muscle to enhance the force of the paralytic muscle. This approach provides good outcomes and is commonly used to treat rectus paralysis. Kamlesh and Dadeya [13] modified Knapp's procedure by transposition of the superior half of equally divided (up to 15 mm) medial and lateral recti for unilateral elevator deficiency and the inferior half after suitable recession or resection for horizontal deviation. The superior half of the medial and lateral recti is employed to address the vertical rectus paralysis, while the residual half tendon width horizontal rectus muscles are recessed-resected to address the horizontal deviation. By only operating on two muscles simultaneously, this approach avoids anterior segment ischemia and spares more muscle tissue for future surgery. Brooks et al. [14] improved transposition of the half of the rectus muscles by shortening one-half the width of the rectus adjacent to the paralytic muscle by 4–8 mm before transposition to the insertion of the paralytic muscle and referred to the technique as augmented partial rectus muscle transposition (APRMT). This technique strengthens the tension of the transposed muscle and provides better corrective effects.

For patients with paralysis of the vertical muscles complicated by a moderate-to-large angle of horizontal strabismus, in addition to correction of the vertical strabismus using rectus transposition, APRMT makes simultaneous horizontal correction possible. In our center, we have used APRMT to correct vertical strabismus and simultaneously performed conventional recession-resection of the remaining half width of the adjacent rectus for the correction of horizontal strabismus.

This purpose of this retrospective study was to report the results of 10 patients with complete vertical rectus paralysis complicated by moderate-to-large angle horizontal strabismus who received APRMT combined with partial horizontal recession-resection.

## 2. Patients and Methods

### 2.1. General Information

The records of patients with complete vertical rectus paralysis complicated by moderate-to-large angle horizontal strabismus who were treated with APRMT combined with partial horizontal recession-resection surgery at the Affiliated Eye, Ear, Nose, and Throat Hospital of Fudan University from April 2011 to August 2012 were retrospectively analyzed. Patients with neurological diseases such as brain tumors and stroke were excluded. Patients with systemic disease and thyroid disease were also excluded. In all cases included in this study, muscular paralysis was due to congenital causes or the result of trauma. This study was approved by the Institutional Review Board of the Eye and ENT Hospital of Fudan University, and because of the retrospective nature the requirement of written informed consent was waived.

In all cases, a detailed medical history was obtained before surgery, and a complete ophthalmological examination was performed. The angle of strabismus at the primary position of the eye and movement of the paralytic muscle were evaluated preoperatively and postoperatively. The prism and alternate cover test was used to measure the angle of deviation from far (6 m) and near (33 cm) distance. Briefly, a prism was placed in front of left eye and the cover was placed alternately in front of each eye, while patient was requested to maintain fixation on far and near small objects with right eye, respectively. No movement of the left eye was the neutralization. The angle of deviation of the left eye was measured, while the right eye was fixating. The same procedure was repeated to determine the angle of deviation of the right eye, while the left eye was fixating. As described by Hong et al. [10] movement of the paralytic muscle was classified into 6 grades with a score from 0 to −5 as follows. Score −5: the eye cannot move in the direction of action of the paralytic muscle; score −4: the eye can just reach the midline while moving in the direction of action of the paralytic muscle; scores −3 to −1: the eye can pass the midline and achieve 25%, 50%, and 75%, respectively, of the rotation from the midline to the maximum rotation in the direction of action of the paralytic muscle; score 0: normal eye movement in the direction of action of the paralytic muscle.

Children 13 years of age and younger underwent general anesthesia. Patients older than 13 years received topical anesthesia 15 min prior to surgery (Benoxil was applied 3 times at intervals of 5 min). They also received 10 mg of nalbuphine hydrochloride given intravenously 10 minutes before surgery. Surgery was initiated when there was no pain response associated with bulbar conjunctiva clamping. Topical anesthetic was applied during surgery as necessary.

### 2.2. Surgical Planning

**Figure 1:**
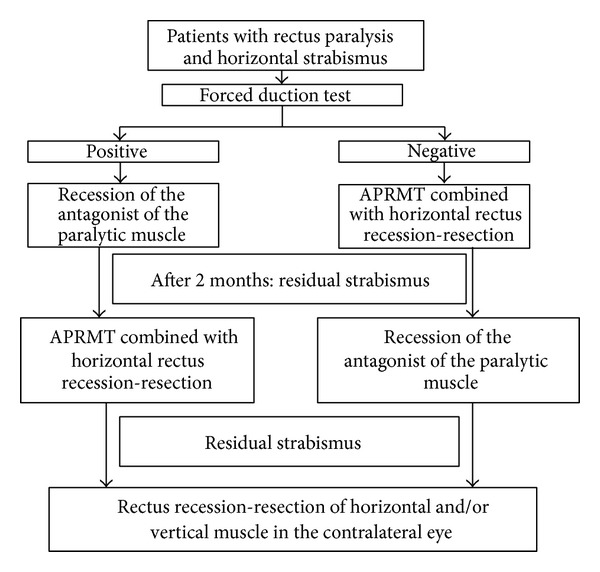
Flowchart of the surgical protocol.

A forced duction test was performed before surgery to determine if the antagonist muscles of the paralytic muscle had any restrictive factors such as contracture. If the forced duction test was positive, antagonist muscle weakening surgery was performed. APRMT + partial horizontal rectus recession-resection was performed 2 months later. If vertical strabismus and/or horizontal strabismus were still present after APRMT + partial horizontal rectus recession-resection, conventional recession-resection of the horizontal and vertical muscles of the contralateral eye was performed at the same time. If the forced duction test was negative, APRMT + partial horizontal rectus recession-resection was performed and, if vertical strabismus and/or horizontal strabismus was still present after APRMT + partial horizontal rectus recession-resection, antagonistic muscle weakening surgery and/or conventional recession-resection of the horizontal and vertical muscles of the contralateral eye was performed 2 months later. A summary of the surgical planning is shown in Figure 1.

### 2.3. APRMT + Partial Horizontal Rectus Recession-Resection

**Figure 2:**
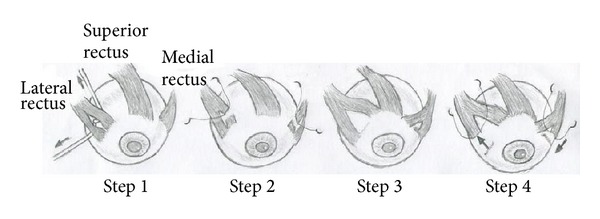
APRMT combined with horizontal muscle recession-resection (using paralysis of the superior rectus muscle as an example). Step  1: the medial rectus muscle and lateral rectus muscle were divided into 2 parts. Step  2: the upper one-half of the medial rectus muscle and lateral rectus muscle was shortened. Step  3: the shortened medial rectus muscle and lateral rectus muscle were transposed to the insertion of the superior rectus muscle on the nasal side and the temporal side. Step  4: recession-resection of the lower one-half of the medial rectus muscle and lateral rectus muscle was performed, and the ends of the recessed and resected muscles were aligned with the 2 original ends.

The rectus transposition technique of Brooks et al. [14] was used. The half width of the 2 recti adjacent to the paralytic muscle was shortened by 3–5 mm before they were transposed to the bilateral insertions of the paralytic muscle. At the same time, the remaining half of the recti underwent conventional recession-resection to correct the horizontal strabismus. The amount of recession-resection was determined according to the angle of the horizontal strabismus. The patients received 2 PD to 3 PD correction per millimeter of recession-resection of the remaining medial rectus muscle and 1 PD to 2 PD correction per millimeter of recession-resection of the remaining lateral rectus muscle. Using paralysis of the superior rectus as an example, the surgical approach was as follows. The conjunctiva and Tenon's capsule were dissected using a Parks incision. The medial rectus, lateral rectus, Krause's membrane, and Whitnall's ligament were dissociated. The medial rectus and lateral rectus were divided into the upper one-half and lower one-half, and 6-0 suture was used to transpose the upper one-half of the medial rectus and lateral rectus to the insertion of the superior rectus on the nasal side and the temporal side, respectively. At the same time, recession-resection of the lower one-half of the medial rectus and lateral rectus was performed according to the angle of the horizontal strabismus. The ends of the recessed and resected muscles were aligned with the 2 original ends (Figure 2). Next, interrupted 8-0 suture was used for closure of Tenon's capsule and the bulbar conjunctiva. After surgery, TobraDex ointment was applied and the eye was bandaged.

Patients were followed up 1 day, 1 week, 1 month, 3 months, and 6 months after surgery.

## 3. Statistical Analysis

Pre- and postoperative deviations and movement scores of the paralytic muscle were represented as means and standard deviations (SD), and the paired* t*-test was used to compare differences. All statistical assessments were two-tailed, and a value of *P* < 0.05 was considered to indicate statistical significance. Statistical analyses were performed using SPSS 18.0 statistics software (SPSS Inc., Chicago, IL, USA).

## 4. Results

Ten patients, 4 male and 6 female, with a mean age of 22.3 ± 13.0 years (range 5 to 39 years) were included in the analysis. Patient characteristics are presented in Table 1. Nine cases were congenital and one case was acquired (due to trauma). There were 4 cases of complete superior rectus paralysis, 3 cases of complete paralysis of the inferior rectus, and 3 cases of monocular elevation deficiency (MED). In all cases, the eye could not pass the midline in the direction of the major action of the paralytic rectus muscle. In addition, in all cases moderate-to-large angle concomitant horizontal strabismus was present. The average duration of follow-up was 7.1 months (range 6 to 8 months), and no patient was lost to follow-up.

Patients 1 and 2 had positive results in the forced duction test and they underwent weakening surgery of the antagonistic muscles first and combined APRMT and partial horizontal rectus recession-resection 2 months later. After the final surgery, the eye position was normal and eye movement was improved. The remaining 8 patients had negative results in the forced duction test; thus they directly underwent APRMT combined with partial horizontal rectus recession-resection. The vertical and horizontal strabismuses of patients 3 to 6 were corrected satisfactorily. Patients 7 through 10 had residual horizontal and/or vertical strabismus, and they underwent weakening surgery of the antagonistic muscles and/or corrective surgery in the contralateral eye for the horizontal and vertical strabismus. The surgical dosing used in the 10 cases is summarized in Table 2.

**Figure 3:**
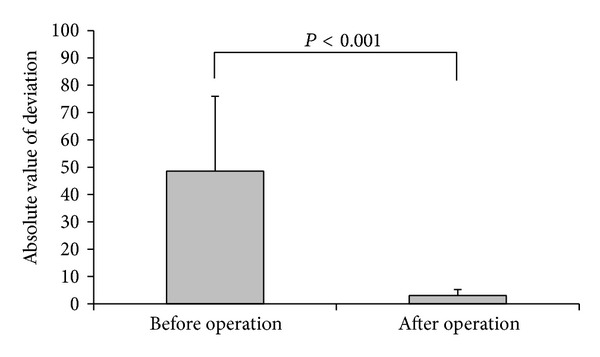
Comparison of the angle of strabismus while looking at far objects (6 m) before APRMT and at the 6-month follow-up. Data are represented as mean with SD for the absolute values of deviations before and after operation. The absolute values before and after deviations were compared using paired* t*-test. *P* < 0.001 indicates significantly different between before and after deviations.

**Figure 4:**
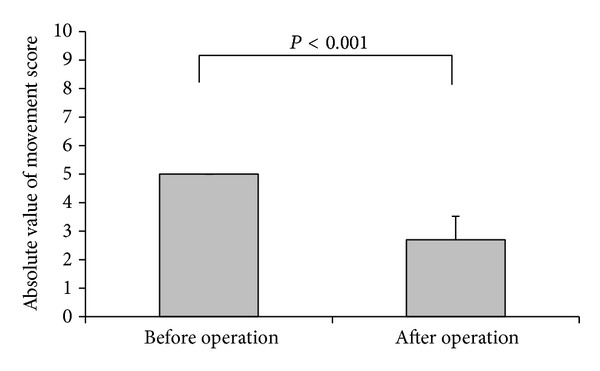
Comparisons of movement scores before surgery and at the 6-month follow-up. Data were represented as mean with SD error bar for in the absolute movement scores before and after operation, respectively. The absolute pre- and postmovement scores were compared using paired* t*-test. *P* < 0.001 indicates significantly different between pre- and postmovement scores.

A comparison of the pre- and postoperative angles of deviation of the paralytic eye of the 10 patients while looking at far objects (6 m) is shown in Figure 3. The mean vertical deviation that APRMT corrected was 21.4 ± 3.7 PD (range: 18 to 30 PD). The horizontal deviation significantly decreased from a preoperative value of 48.5 ± 27.4 PD to a value of 3.0 ± 2.3 PD 6 months postoperatively (*P* < 0.001). Six patients (patients 3, 4, 5, 6, 8, and 10) without antagonistic muscle weakening surgery had no changes in the degree of horizontal and vertical strabismus between one week and six months. Four patients (patients 1, 2, 7, and 9) with antagonistic muscle weakening surgery had no changes in the degree of horizontal strabismus but had 5 PD changes of vertical strabismus between one week and six months. The pre- and postoperative movement scores of the paralytic muscles are shown in Figure 4. The movement score decreased from a value of −5 ± 0 preoperatively to a value of −2.7 ± 0.8 at 6 months postoperatively (*P* < 0.001).

**Figure 5:**
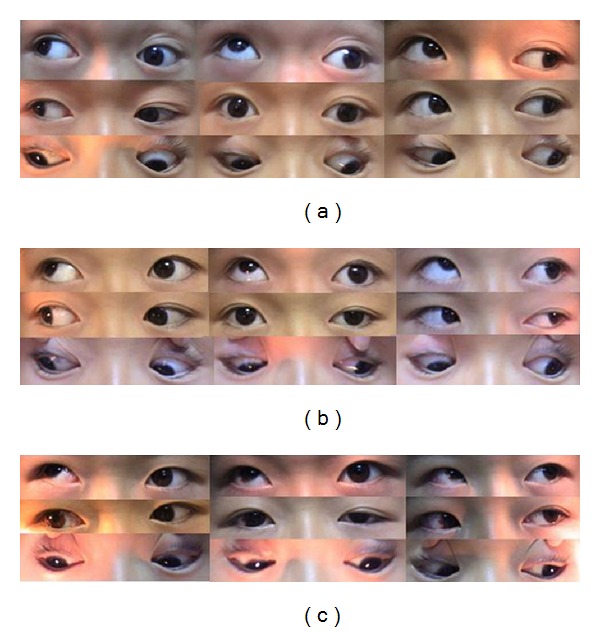
(a) Preoperative images for patient 10. Before surgery insufficient supraduction of the left eye complicated by exotropia was noted. The supraduction of the left eye could not pass the midline. (b) After APRMT combined with partial horizontal rectus recession-resection on the paralytic eye (left eye), insufficient supraduction of the left eye was still present, but the vertical strabismus was corrected at the primary position. The movement of the paralytic eye was improved and exotropia was still present. (c) After horizontal rectus recession-resection on the contralateral (right) eye 2 months later, both horizontal and vertical strabismuses were corrected at the primary position. The movement of the paralytic eye was improved, although the supraduction was still insufficient.

As a representative case, the pre- and postoperative deviations of patient 10 are summarized in Table 3, and pre- and postoperative images of patient 10 are shown in Figure 5.

## 5. Discussion

In this study, we reported the results of 10 patients with vertical rectus paralysis complicated by moderate-to-large angle horizontal strabismus who received APRMT combined with partial horizontal recession-resection. The mean vertical deviation that APRMT corrected was 21.4 ± 3.7 PD (range: 18 PD to 30 PD). The horizontal deviations of strabismus were decreased from a value of 48.5 ± 27.4 PD preoperatively to a value of 3.0 ± 2.3 PD 6 months postoperatively. The movement score of the paralytic muscles was also significantly improved from a preoperative value of −5 ± 0 to a value of −2.7 ± 0.8 postoperatively. No unexpected horizontal or vertical strabismus occurred. A standard amount of half horizontal rectus recession-resection was used; no excessive surgical amount was adopted in any patient. No surgical complications occurred.

Because the blood supply of the anterior segment tissues comes from the anterior ciliary artery and 7 ciliary arteries enter the eye through 4 extraocular muscles, surgery involving more than 2 recti is generally avoided to decrease the risk of anterior segment ischemia [15]. When vertical muscle paralysis is accompanied by horizontal strabismus, if transposition of the whole horizontal recti is performed, the horizontal strabismus can only be corrected in the unaffected eye. The use of Brooks' APRMT makes partial or complete correction of horizontal strabismus in the same eye possible.

In this study, APRMT was combined with partial horizontal rectus recession-resection. The surgical approach in the vertical direction remained the same in patients with combined horizontal strabismus, and the untransposed half of the muscle underwent recession-resection surgery. Since only two recti are involved, the risk of anterior segment ischemia is low; however, both horizontal and vertical strabismus can be corrected. During suturing, both sides of the muscles were aligned with the original insertion of the muscle so that the anatomical position of the muscle was restored. This technique can address the problem of horizontal and vertical strabismus simultaneously, the number of muscles involved in the surgery is reduced, and avoidance of a second surgery is possible [16]. Of particular note, the preoperative forced duction test is very important for the determination of the surgical approach. A positive forced duction test indicates contracture of the muscle antagonistic to the paralyzed muscle, therefore, conventional weakening surgery of the antagonistic muscles should be performed first to fully release the spastic antagonistic muscle and APRMT can then be performed 2 months later, which is enough time for healing to occur.

In case number 10, a typical pediatric patient, APRMT corrected the vertical strabismus and recession-resection of half of the horizontal rectus partially corrected the horizontal strabismus. Surgery was then performed on the contralateral eye to correct the residual horizontal strabismus 2 months later. The strabismus at the primary eye position was basically corrected, and the compensatory head position disappeared. The supraduction of the left eye was still insufficient but was improved, and the effects were stable at the 6-month follow-up.

Because of the complexity of paralytic strabismus and individual differences, surgical correction is nonquantitative [1, 3]. Knapp [12] reported the results of 14 patients with full tendon rectus transposition, and with follow-up of 4 to 5 years the average correction was 39 PD. The corrective effect of half rectus transposition has been rarely reported. Lu [17] reported that half tendon rectus translocation could obtain 30 PD correction and found that the correction amount was related to the preoperative angle of strabismus, strength of the transposed muscle, and length of time of paralysis. In this study, the median vertical correction was 21.4 PD after APRMT. It is noteworthy that after APRMT, the angle of deviation may change with time. Kamlesh and Dadeya [13] reported that when simple half tendon rectus transposition was used to correct vertical strabismus, patients had residual vertical strabismus after surgery and the average reduction in the residual vertical deviation was 8.5 PD from 6 weeks to 21 months after surgery. The technique we have described is an improvement of that described by Kamlesh and Dadeya [13], which combined their procedure with that of Brooks et al. [14]. The muscle to be transposed is first shortened by 3–5 mm, which helps to provide more tension and a more anatomical position. In this technique, the augmented partial rectus muscle transposition is employed to address the vertical rectus paralysis, while the residual half tendon width horizontal rectus muscles are recessed/resected to address the horizontal deviation. By only operating on two muscles simultaneously, this approach avoids anterior segment ischemia and spares more muscle tissue for future surgery, if necessary. Four of ten patients achieved satisfactory outcomes after only one surgery.

For the correction of horizontal strabismus, the patients received 2 PD to 3 PD correction per millimeter of recession-resection of the remaining medial rectus muscle and 1 PD to 2 PD correction per millimeter of recession-resection of the remaining lateral rectus muscle. In this study, combined half horizontal rectus recession-resection corrected the horizontal strabismus in 4 patients.

The primarily limitations of this study are the small number of cases and the patient population was heterogeneous with both adult and pediatric cases included. APRMT strabismus surgery is successful, but it still needs long-term follow-up to evaluate the final outcomes.

## 6. Conclusions

For patients with vertical rectus paralysis combined with moderate-to-large angle horizontal strabismus, combined APRMT and partial horizontal rectus recession-resection is safe and effective for correcting vertical and horizontal strabismus.

## Figures and Tables

**Table 1:** Patient characteristics.

Patient	Age (y)	Sex	Diagnosis	Angle of strabismus (PD)		Movement score of paralytic muscle		Follow-up (mo)
				Preoperative	6 months Postoperative^a^	Preoperative	Postoperative	
1	39	Female	RIRP + XT	XT 45 RHT 50	X 3 RH 2	−5	−2	6
2	36	Female	RIRP + XT	XT 35 RHT 80	XT 7 RHT 6	−5	−2	7.5
3	24	Female	MED + XT	XT 20 RH 20	0	−5	−2	7.5
4	35	Female	MED + XT	XT 30 LHT 25	X 2 LH 2	−5	−2	6
5	21	Female	LIRP + XT	XT 30 LHT 25	X 2 LH 2	−5	−4	8
6	32	Male	RSRP + XT	XT 25 LHT 30	X 1	−5	−3	8
7	17	Female	LSRP + XT	XT 80 RHT 35	X 5 RH 3	−5	−3	8
8	5	Male	MED + XT	XT 40 LHT 20	X 2	−5	−2	8
9	5	Male	RSRP + ET	ET 90 LHT 60	ET 6 LHT 4	−5	−3	6
10	9	Male	LSRP + XT	XT 90 RHT 25	X 2 RH 1	−5	−4	6

RIRP: right inferior rectus paralysis; LIRP: left inferior rectus muscle paralysis; RSRP: right superior rectus paralysis; LSRP: left superior rectus paralysis; MED: monocular elevation deficiency; XT: exotropia; ET: esotropia; X: exophoria; RHT: right hypertropia; LHT: left hypertropia; RH: right hyperphoria; LH: left hyperphoria; PD: prism diopter.

^a^In case 3, 6, and 8 postoperative vertical strabismus was not present.

**Table 2:** Surgical dosing used in the 10 cases.

Patient	Surgical plan	
	First stage	Second stage
1	RSR rec 8 mm	APRMT + partial RMR res 6 mm + RLR rec 8 mm
2	RSR rec 8 mm	APRMT + partial RMR res 6 mm + RLR rec 6 mm
3	APRMT + partial LMR res 4.5 mm + LLR rec 5 mm	/
4	APRMT + partial RMR res 6 mm + RLR rec 5 mm	/
5	APRMT + partial LMR res 6 mm + LLR rec 6 mm	/
6	APRMT + partial RMR res 4 mm + RLR rec 5 mm	/
7	APRMT + partial LMR res 6 mm + LLR rec 8 mm	LIR rec 2 mm + RMR res 3 mm + RLR rec 4 mm
8	APRMT + partial RMR res 6 mm + RLR rec 8 mm	LMR res 2 mm
9	APRMT + partial RMR rec 6 mm + RLR res 8 mm	RIR rec 6 mm + LMR rec 5 mm + LLR res 6 mm
10	APRMT + partial LMR res 6 mm + LLR rec 8 mm	RMR res 4 mm + RLR rec 5.5 mm

APRMT: augmented partial rectus muscle transposition; RSR: right superior rectus muscle; RIR: right inferior rectus muscle; LIR: left inferior rectus muscle; RMR: right medial rectus muscle; RLR: right lateral rectus muscle; LMR: left medial rectus muscle; LLR: left lateral rectus muscle; res: resection; rec: recession.

/ indicates no second procedure performed.

**Table 3:** Angles of strabismus of the unaffected eye in patient 10 before and after surgery.

Time	Distance	
	33 cm	6 m
Before operation	XT 75 RHT 25	XT 90 RHT 25
After APRMT + partial horizontal rectus recession-resection	XT 50 RH 5	XT 50 RH 5
1 week after horizontal rectus recession-resection on the contralateral (right) eye	X 2 RH 2	X 3 RH 3
6 months after horizontal rectus recession-resection on the contralateral (right) eye	X 2 RH 2	X 2 RH 1

APRMT: augmented partial rectus muscle transposition; XT: exotropia; X: exophoria; RHT: right hypertropia; RH: right hyperphoria.
